# Whole-body MRI versus an FDG-PET/CT-based reference standard for staging of paediatric Hodgkin lymphoma: a prospective multicentre study

**DOI:** 10.1007/s00330-020-07182-0

**Published:** 2020-09-03

**Authors:** Suzanne Spijkers, Annemieke S. Littooij, Thomas C. Kwee, Nelleke Tolboom, Auke Beishuizen, Marrie C. A. Bruin, Sjoerd G. Elias, Tim van de Brug, Goya Enríquez, Constantino Sábado, Elka Miller, Claudio Granata, Charlotte de Lange, Federico Verzegnassi, Mary-Louise C. Greer, Bart de Keizer, Rutger A. J. Nievelstein

**Affiliations:** 1 Department of Radiology and Nuclear Medicine, University Medical Center Utrecht/Wilhelmina Children’s Hospital, Utrecht University, Heidelberglaan 100, 3584 CX Utrecht, The Netherlands; 2 Princess Máxima Center for Paediatric Oncology, Utrecht, The Netherlands; 3 Medical Imaging Center, Department of Radiology, University Medical Centre Groningen, University of Groningen, Groningen, The Netherlands; 4 Department of Paediatric Oncology/Haematology, Erasmus Medical Center-Sophia Children’s Hospital, Rotterdam, The Netherlands; 5 Julius Center for Health Sciences and Primary Care, University Medical Center Utrecht, Utrecht University, Utrecht, The Netherlands; 6 Department of Epidemiology and Biostatistics, Amsterdam University Medical Centers, VUmc, Amsterdam, The Netherlands; 7 Institut de Recerca Vall d’Hebron, Barcelona, Spain; 8 Department of Paediatric Oncology and Haematology, University Hospital Vall d’Hebron, Barcelona, Spain; 9 Department of Medical Imaging, CHEO, University of Ottawa, Ottawa, Canada; 10 Department of Paediatric Radiology, Institute for Maternal and Child Health IRCCS Burlo Garofolo, Trieste, Italy; 11 Department of Diagnostic Imaging and Intervention, Oslo University Hospital, Rikshospitalet, Oslo, Norway; 12 Oncohematology Unit, Institute for Maternal and Child Health IRCCS Burlo Garofolo, Trieste, Italy; 13 Department of Diagnostic Imaging, The Hospital for Sick Children, Department of Medical Imaging, University of Toronto, Toronto, Ontario Canada

**Keywords:** Whole-body imaging, Child, Diffusion magnetic resonance imaging, Hodgkin disease, Neoplasm staging

## Abstract

**Objectives:**

To assess the concordance of whole-body MRI (WB-MRI) and an FDG-PET/CT-based reference standard for the initial staging in children with Hodgkin lymphoma (HL)

**Methods:**

Children with newly diagnosed HL were included in this prospective, multicentre, international study and underwent WB-MRI and FDG-PET/CT at staging. Two radiologists and a nuclear medicine physician independently evaluated all images. Discrepancies between WB-MRI and FDG-PET/CT were assessed by an expert panel. All FDG-PET/CT errors were corrected to derive the FDG-PET/CT-based reference standard. The expert panel corrected all reader errors in the WB-MRI DWI dataset to form the intrinsic MRI data. Inter-observer agreement for WB-MRI DWI was calculated using overall agreement, specific agreements and kappa statistics. Concordance for correct classification of all disease sites and disease stage between WB-MRI (without DWI, with DWI and intrinsic WB-MRI DWI) and the reference standard was calculated as primary outcome. Secondary outcomes included positive predictive value, negative predictive value and kappa statistics. Clustering within patients was accounted for using a mixed-effect logistic regression model with random intercepts and a multilevel kappa analysis.

**Results:**

Sixty-eight children were included. Inter-observer agreement between WB-MRI DWI readers was good for disease stage (*κ* = 0.74). WB-MRI DWI agreed with the FDG-PET/CT-based reference standard for determining disease stage in 96% of the patients versus 88% for WB-MRI without DWI. Agreement between WB-MRI DWI and the reference standard was excellent for both nodal (98%) and extra-nodal (100%) staging.

**Conclusions:**

WB-MRI DWI showed excellent agreement with the FDG-PET/CT-based reference standard. The addition of DWI to the WB-MRI protocol improved the staging agreement.

**Key Points:**

*• This study showed excellent agreement between WB-MRI DWI and an FDG-PET/CT-based reference standard for staging paediatric HL.*

*• Diffusion-weighted imaging is a useful addition to WB-MRI in staging paediatric HL.*

*• Inter-observer agreement for WB-MRI DWI was good for both nodal and extra-nodal staging and determining disease stage.*

**Electronic supplementary material:**

The online version of this article (10.1007/s00330-020-07182-0) contains supplementary material, which is available to authorized users.

## Introduction

Hodgkin lymphoma (HL) is amongst the most prevalent childhood cancers, and it is the most common type of cancer in adolescents [1]. After diagnosis, determining the extent of disease (staging) is important for the choice of treatment. The Lugano staging system that is used for staging HL distinguishes four disease stages, with (B) or without (A) disease symptoms or E-lesions (E, extra-nodal extension) [2, 3]. The standard treatment consists of chemotherapy and radiotherapy. Limited-stage disease needs less treatment than advanced-stage disease. Radiotherapy can be omitted based on ^18^F-fluorodeoxyglucose positron emission tomography (FDG-PET)/computed tomography (CT) response measurement. Nowadays, the imaging modality that is considered the reference standard for staging HL is FDG-PET/CT [2, 4, 5]. Unfortunately, FDG-PET/CT is accompanied by the exposure to ionizing radiation. The overall paediatric HL survival rates are around 95% [1, 6]. Therefore, children with HL will generally have a long life expectancy after their treatment, which implicates a long time frame in which long-term side effects of their radiation exposure during diagnosis and treatment can occur. The administered ionizing radiation dose is 5 millisievert (mSV) per FDG-PET/CT in the University Medical Center Utrecht, depending on whether a low-dose CT or a high-dose contrast-enhanced CT is used. In other centres, the ionizing radiation doses are reportedly higher, up to 23 ± 11 mSV per FDG-PET/CT, especially since a contrast-enhanced CT is still part of standard procedures in many hospitals [7]. Since during staging and follow-up repeated imaging is required, the radiation dose accumulates to even higher levels. Combined with the increased susceptibility of children to the effects of ionizing radiation exposure [8], children with HL are at risk of developing secondary malignancies during their further lifetime [9–14].

Whole-body magnetic resonance imaging with diffusion-weighted imaging (WB-MRI with DWI) is a radiation-free method which allows imaging of the body with excellent soft tissue contrast in a single examination and could therefore be an attractive alternative to FDG-PET/CT for the staging of HL in children [15–18]. With the addition of DWI to the WB-MRI protocols, it is suggested that not only anatomical but functional information is provided as well, offering a possible surrogate to the functional information provided with FDG-PET/CT [19]. The evidence for the use of WB-MRI with DWI for staging HL in children, although increasing, is still limited [16, 17, 20, 21]. The aim of this study was to compare the concordance of WB-MRI (including DWI) and FDG-PET/CT for initial staging in children with Hodgkin lymphoma in order to contribute to the development of evidence-based ‘radiation reduced’ imaging protocols in paediatric Hodgkin lymphoma.

## Methods

A total of 10 hospitals participated in this prospective international cohort study: University Medical Center Utrecht, University Children’s Hospital Vall d’Hebron Barcelona, Amsterdam University Medical Centers, CHEO Ottawa, Giannina Gaslini Children’s Hospital Genova, Erasmus Medical Center–Sophia Children’s Hospital Rotterdam, Materno Infantile Burlo Garofolo Trieste, Oslo University Hospital, Rikhospitalet and The Hospital for Sick Children Toronto. The local institutional review boards of all participating centres approved this prospective study. Depending on the age of the participant, written informed consent was obtained from all study participants and/or their parents or guardians.

### Study population

All European patients were included in the Euronet PHL-C1 trial (First International Inter-group Study for Classical Hodgkin’s Lymphoma in Children and Adolescents) [22, 23]. Inclusion criteria were age 7–18 years with newly diagnosed, histologically proven HL. All patients were included between March 2012 and January 2016. Exclusion criteria were general contraindications for MRI (e.g. pacemaker, metallic implant and claustrophobia), previous malignancies, and breastfeeding or pregnancy.

### Procedures

Patients underwent both an FDG-PET/CT and WB-MRI at staging before start of treatment. The FDG-PET/CT was made as part of standard clinical care and WB-MRI was always performed within 15 days of the FDG-PET/CT (median 1.00 days, interquartile range (IQR), 4.00). Full descriptions of the WB-MRI and FDG-PET/CT protocols used by all participating centres are provided in the supplementary material file. WB-MRI sequence parameters are shown in supplementary table 1. All images were de-identified and collected for review.

### Whole-body MRI image interpretation

The de-identified WB-MR images were analysed by two independent radiologists (R.A.J.N. and T.C.K. with 25 and 10 years of MRI experience, respectively) using OsiriX Lite Medical Imaging Software (Pixmeo) or Horos (Horos Project). The readers were aware of the diagnosis of HL, but had no access to other information such as clinical data and other imaging findings. Analyses and scoring were performed using a standardized form based on the Euronet PHL C1 trial [22, 23]. The readers evaluated first the WB-MRI without DWI (T1-weighted and T2-weighted only) and second the WB-MRI including DWI immediately thereafter. Disease presence was scored for 10 nodal and all possible extra-nodal regions (e.g. thoracic, abdominal, central nervous system and musculoskeletal sites). Nodal regions were cervical, axillary, infraclavicular, mediastinal, hilar, spleen, para-aortic, mesenteric, para-iliac and inguinal. The relevant extra-nodal regions were lung, liver and bone marrow. Table 1 summarizes the criteria for involvement of the different nodal and extra-nodal regions. Finally, the disease stage was determined for each reading [24]. Discrepancies between the datasets from both WB-MRI readers were solved by a third reader (A.S.L, radiologist, with 15 years of MRI experience) to form the final consensus WB-MRI datasets (both with and without DWI).

**Table 1:** Criteria for nodal and extra-nodal involvement at WB-MRI

Site	Definition
Nodal	
Lymph nodes	Longest diameter > 15 mm or shortest diameter > 10 mm
Spleen	Discrete nodules with focal low signal on T2-weighted MRI^1^ and DWI, or enlargement (vertical maximum diameter > 13 cm)
Extra-nodal	
Liver	Nodules, moderate hyperintense T2-weighted images, separate from adjacent lymphatic mass
Lung	Discrete lesion > 10 mm
Bone marrow	Hypointense T1-weighted and hyperintense T2-weighted combined with focal restricted diffusion (as compared to surrounding normal bone marrow)
E lesion	Disease infiltration into extra-lymphatic structure or organ that is adjacent to a lymph node mass

^1^Littooij A.S. et al, *Acta Radiologica* 2015

### FDG-PET/CT image interpretation

The de-identified FDG-PET/CT images were analysed by a nuclear medicine physician (B.d.K., with 15 years of FDG-PET/CT experience) using OsiriX Lite Medical Imaging Software. The reader was blinded to clinical data and other imaging findings not related to the lymphoma diagnosis. Disease presence was scored either positive (e.g. FDG uptake above uptake in mediastinum and/or liver) or negative for 10 nodal and all extra-nodal stations. Disease stage was reported [24].

### Expert panel: forming the reference standard and intrinsic WB-MRI dataset

An independent expert panel reviewed all discrepancies between consensus WB-MRI and FDG-PET/CT scoring results. The expert panel consisted of a radiologist (A.S.L.) and a nuclear medicine physician (N.T., with 9 years of FDG-PET/CT experience). The expert panel had access to all the available clinical and imaging information. All discrepancies were assessed and labelled as reader error or intrinsic error. Reader errors were being caused by failure of the reader to detect the abnormality (perceptual error) or by an incorrect interpretation of an abnormal finding (interpretation error). Intrinsic errors could either be due to limitations of the imaging acquisition or technique (e.g. if the abnormality was outside the field of view or if the error was caused by severe artefacts). Reader errors were corrected for WB-MRI including DWI to form the intrinsic WB-MRI reading. The FDG-PET/CT-based reference standard was formed by correcting all FDG-PET/CT reader and intrinsic errors.

### Statistical analysis

The statistical analyses were performed using the Statistical Package for the Social Sciences (SPSS), version 25.0, and the R statistical software package version 3.5.1 (R Development Core Team).

Concordance between WB-MRI without DWI, WB-MRI with DWI and intrinsic WB-MRI and the FDG-PET/CT-based reference standard was assessed by calculating total agreement, positive predictive values (PPV), negative predictive values (NPV) and Cohen’s kappa statistics. Those were calculated between WB-MRI and the reference standard for lymphoma detection per patient (disease stage) and for presence/absence of disease in the separate nodal and extra-nodal stations as well as for the combined nodal and extra-nodal stations. Kappa values for staging agreement with and without DWI were compared and tested as proposed by Vanbelle to determine the additional value of DWI [25]. A *p* value < 0.05 was considered statistically significant.

Sensitivity and specificity of WB-MRI without DWI, WB-MRI with DWI and intrinsic MRI for staging were calculated against the reference standard.

Inter-observer agreement between the two WB-MRI readers was assessed using percentages of observed and specific agreement (expressing the agreements for positive and negative ratings separately) and kappa statistics [26].

For all analyses of the combined nodal and extra-nodal stations, clustering within patients had to be considered. Multilevel analyses were performed as proposed by Vanbelle et al [25] for the kappa statistics. For observed agreement, PPV and NPV a mixed-effect logistic regression model was used, taking clustering within patients into account using random intercepts.

For all Cohen’s kappa analyses, the kappa values were interpreted as poor (*κ* < 0.2), fair (*κ* 0.2–0.4), moderate (*κ* > 0.4–0.6), good (*κ* > 0.6–0.8) and excellent (*κ* > 0.8) [27].

## Results

### Patient characteristics

Seventy-six patients were found eligible and were prospectively included between 2012 and 2016. Eight patients were excluded due to no informed consent (*n* = 1), incomplete MRI study (*n* = 2) or logistic circumstances (*n* = 5; reasons included patient too sick to undergo WB-MRI (*n* = 2), no certain HL diagnosis at scheduled time of WB-MRI (*n* = 1), WB-MRI scheduled at the same time as another examination (*n* = 2)). All remaining 68 patients underwent WB-MRI and FDG-PET/CT for staging. The baseline characteristics, including age, gender, HL subtype and disease stage, are shown in Table 2.

**Table 2:** Baseline characteristics

	*N* (%) *n* = 68
Age (years)	
Mean (SD)	14 (2.3)
Range	7–17
Gender	
Male	33 (48.5)
Female	35 (51.5)
Hodgkin lymphoma subtype	
Classical	
Nodular sclerosing HL	35 (51.5)
HL, lymphocyte rich	6 (8.8)
HL, mixed cellularity	5 (7.4)
HL, lymphocyte depleted	0 (–)
Classical HL, not otherwise specified	22 (32.3)
Nodular lymphocyte predominant	0 (–)
Disease stage	
I	7 (10.3)
II	38 (55.9)
III	11 (16.2)
IV	12 (17.6)

*HL* Hodgkin lymphoma, *SD* standard deviation

### Inter-observer agreement, WB-MRI

Table 3 summarizes the inter-observer agreement between both WB-MRI readers. Overall agreement between readers for disease stage (limited versus advanced disease) was 88% (60/68, 95% CI 0.78–0.93) and kappa agreement was good (*κ* 0.74, 95% CI 0.58–0.91). The specific agreement on the positive ratings was overall lower compared to the specific agreement on the negative ratings, indicating that readers were more likely to agree on negative than positive rating for disease presence. The lymph node stations with the lowest agreements were hilar (74%, 50/68) and infraclavicular (77%, 52/68). For all other stations, the observed agreements were ≥ 90%.

**Table 3:** Inter-observer agreement WB-MRI DWI for initial staging of paediatric Hodgkin lymphoma

	Observed agreement (total) (*n*/*n*, 95% CI)	Specific agreement positive ratings (95% CI)	Specific agreement negative ratings (95% CI)	Kappa (95% CI)
Disease stage (stage I/II vs III/IV)	0.88 (60/68, 0.78–0.93)	0.83 (0.71–0.92)	0.91 (0.84–0.96)	0.74 (0.58–0.91)
All sites combined	0.92 (801/884, 0.89–0.94)	0.83 (0.79–0.87)*	0.93 (0.92–0.95)*	0.77 (0.72–0.81)
All nodal sites combined	0.91 (607/680, 0.87–0.93)	0.83 (0.79–0.88)*	0.92 (0.90–0.94)*	0.76 (0.70–0.81)
All extra-nodal sites combined	0.95 (194/204, 0.91–0.97)	0.75 (0.56–0.87)*	0.97 (0.95–0.99)*	0.72 (0.57–0.87)
Nodal sites				
Cervical	0.94 (64/68, 0.86–0.98)	0.97 (0.93–0.99)	0.67 (0.37–0.86)	0.64 (0.31–0.96)
Axillary	0.94 (64/68, 0.86–0.98)	0.89 (0.78–0.95)	0.96 (0.91–0.98)	0.85 (0.72–0.99)
Infraclavicular	0.77 (52/68, 0.65–0.85)	0.53 (0.32–0.71)	0.84 (0.75–0.91)	0.38 (0.13–0.62)
Mediastinal	0.96 (65/68, 0.88–0.98)	0.98 (0.95–0.99)	0.73 (0.44–0.89)	0.71 (0.39–1.00)
Hilar	0.74 (50/68, 0.62–0.83)	0.64 (0.47–0.78)	0.79 (0.68–0.88)	0.46 (0.28–0.65)
Para-aortic	0.91 (62/68, 0.82–0.96)	0.80 (0.63–0.91)	0.94 (0.89–0.98)	0.75 (0.56–0.93)
Spleen	0.90 (61/68, 0.80–0.95)	0.63 (0.38–0.82)	0.94 (0.89–0.97)	0.58 (0.31–0.85)
Mesenteric	0.90 (61/68, 0.80–0.95)	0.36 (0.07–0.86)	0.94 (0.89–0.97)	0.32 (0.00–0.69)
Para-iliac	0.96 (65/68, 0.88–0.98)	0.82 (0.63–0.93)	0.97 (0.94–0.99)	0.80 (0.58–1.00)
Inguinal femoral	0.93 (63/68, 0.93–0.97)	NA	0.96 (0.92–0.98)	NA
Extra-nodal sites				
Abdominal	1.00 (68/68, 0.95–1.00)	NA	1.00 (0.99–1.00)	1.00 (NA)
Thoracic	0.90 (61/68, 0.80–0.95)	0.67 (0.43–0.84)	0.94 (0.89–0.97)	0.61 (0.34–0.87)
Bone marrow	0.96 (65/68, 0.88–0.98)	0.84 (0.66–0.94)	0.97 (0.94–0.99)	0.82 (0.62–1.00)

*CI* confidence interval, *NA* not applicable

*No multilevel analysis available

### Reference standard and intrinsic WB-MRI data

**Fig. 1:**
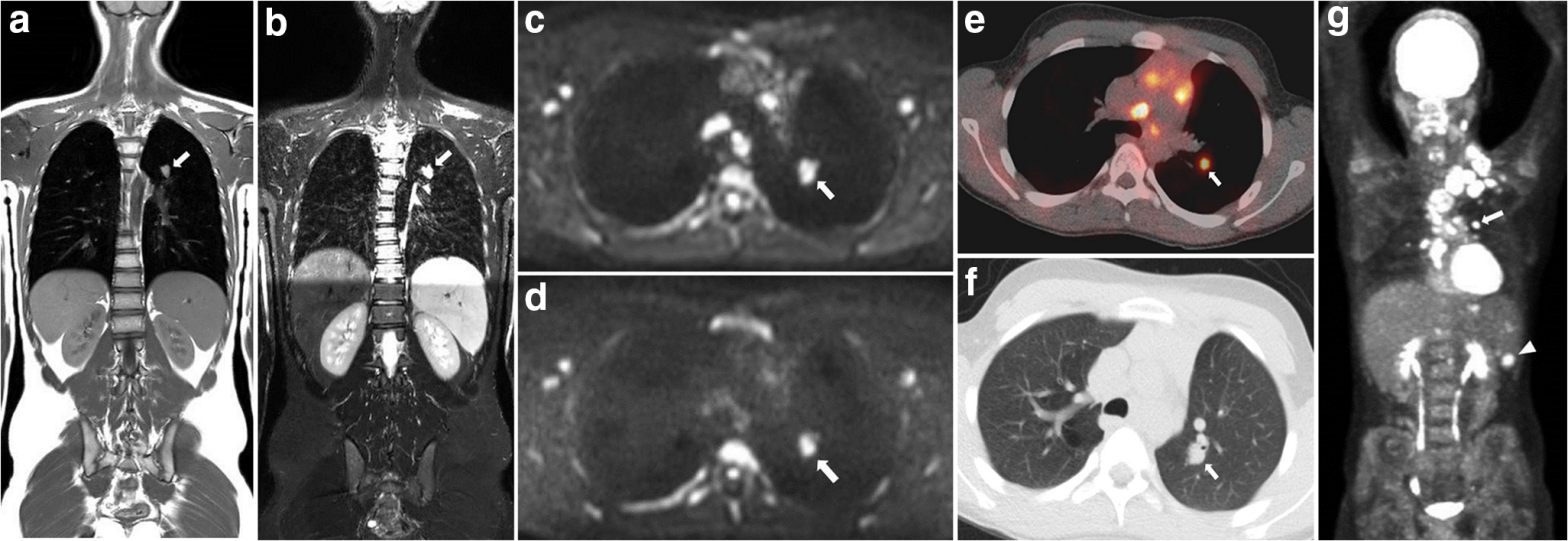
A 15-year-old boy with stage IV Hodgkin lymphoma. Example of an interpretation error by the FDG-PET/CT reader. Involvement of the lung was misinterpreted as hilar involvement. Coronal T1-weighted (**a**) and T2-weighted (**b**) MRI show the involvement of the lung (arrows). At axial DWI (b100 (**c**) and b800 (**d**), DWI restricted diffusion is seen (arrows). At axial FDG-PET/CT (**e**) and CT (**f**), the lesion is seen as well (arrows). Coronal maximum intensity projection (MIP) of the FDG-PET/CT (**g**) shows involvement of the lung (arrow) and multiple lymph node stations including cervical, mediastinal, hilar and the spleen (arrowhead). Those were all seen at WB-MRI as well.

**Fig. 2:**
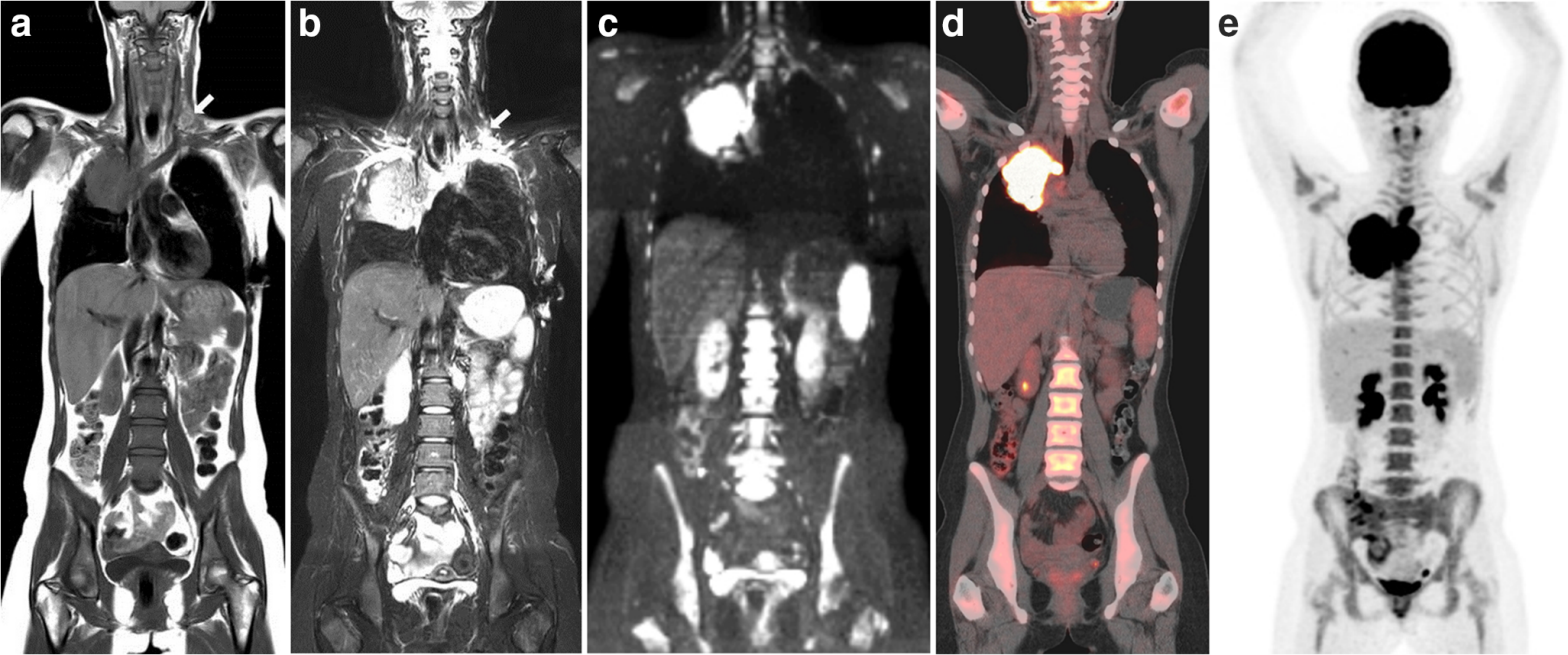
A 17-year-old girl with stage IIE Hodgkin lymphoma. Example of an interpretation error by the WB-MRI readers due to a lack of clinical data. A lesion due to the placement of a central venous catheter was mistaken for presence of Hodgkin lymphoma (arrows). Furthermore, an E-lesion in the lung originating from the mediastinum is present. Coronal T1-weighted (**a**) and T2-weighted (**b**) MRI, high *b* value DWI (b800) (**c**), coronal FDG-PET/CT (**d**) and coronal maximum intensity projection (MIP) of the FDG-PET/CT (**e**) are shown. Note that the supraclavicular lesion was not seen at the FDG-PET/CT MIP (**e**)

The expert panel identified a total of 43 discrepant disease sites (5% of all examined disease sites) between consensus WB-MRI and FDG-PET/CT (31 nodal and 12 extra-nodal sites). Twenty-three FDG-PET/CT reader errors in 17 patients were corrected (19 perception errors and 4 interpretation errors), and no intrinsic FDG-PET/CT errors were identified. Figure 1 shows an example of an interpretation error by the FDG-PET/CT reader. To obtain the intrinsic WB-MRI dataset, the expert panel identified and corrected 9 WB-MRI reader errors in 8 patients. The corrected errors were 8 perception errors and 1 interpretation error. The perception errors were located in the following stations: spleen (3 patients), axillary, hilar, para-iliac, para-aortic and mediastinal. The error of interpretation that was corrected was caused by misinterpretation of a cervical lesion due to the placement of a central venous catheter (lack of clinical information) (Fig. 2).

### Consensus WB-MRI versus FDG-PET/CT-based reference standard

**Fig. 3:**
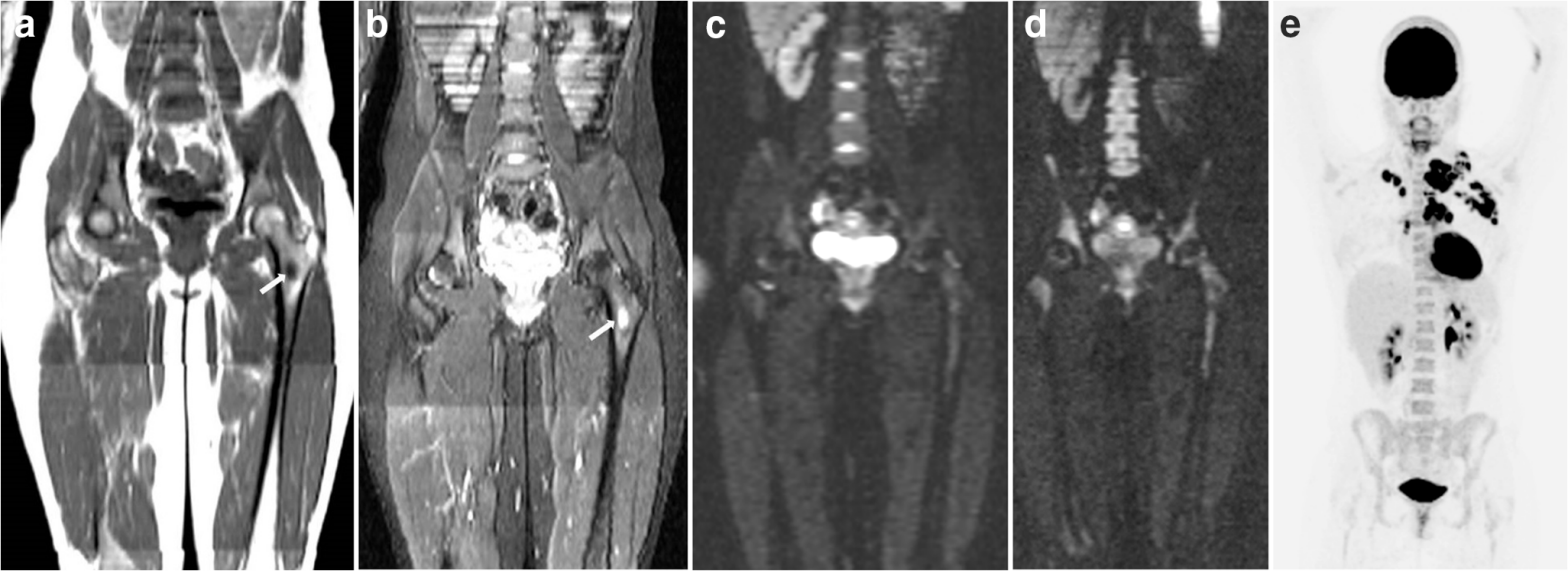
A 17-year-old girl with stage II Hodgkin lymphoma. Example in which the addition of DWI improved the agreement with the reference standard. Coronal T1-weighted MRI (**a**) shows a hypointense lesion in the left femur (arrow) that was interpreted as HL bone marrow involvement by the WB-MRI readers. At coronal T2-weighted MRI (**b**), the lesion appears as a hyperintensity (arrow). Coronal DWI (b100 (**c**) and 800 (**d**) shows no restricted diffusion in the left femur, and thus, no bone marrow involvement was reported by the readers for the WB-MRI reading including DWI. Coronal maximum intensity projection (MIP) of the FDG-PET (**e**) shows involvement of the cervical, axillary, infraclavicular and mediastinal lymph node stations, but no signs of bone marrow involvement are present

The additional value of DWI to T1-weighted and T2-weighted images in staging paediatric HL was assessed by comparing the consensus WB-MRI dataset with and without DWI to the FDG-PET/CT-based reference standard (Table 4). Overall, the agreement for disease stage in each patient improved with the addition of DWI from 88.2% (60/68) to 95.6% (65/68). Kappa values for determining the correct stage (I/II versus III/IV) improved from 0.81 (95% CI 0.67–0.95) to 0.94 (95% CI 0.84–1.00), with a *p* value of 0.036. A total of 10 discrepancies in 9 patients was found when comparing the datasets with and without DWI (Table 5). These differences were found in the following stations: hilar (3 patients), para-aortic (2 patients), mesenteric (2 patients), liver (1 patient) and bone marrow (2 patients) (Fig. 3). Staging results improved in 5 out of 9 patients with the addition of DWI as compared to the FDG-PET/CT-based reference standard. Out of these 5 patients, WB-MRI without DWI would have resulted in upstaging in three patients, whereas it would have resulted in downstaging in two patients.

**Table 4:** Agreement rate, positive predictive value (PPV), negative predictive value (NPV) and Cohen’s kappa for nodal and extra-nodal staging when comparing WB-MRI to the FDG-PET/CT-based reference standard

	Consensus MRI without DWI				Consensus MRI with DWI				Intrinsic WB-MRI DWI*			
	Observed agreement (*n*/*n*, 95% CI)	PPV % (95% CI)	NPV % (95% CI)	Kappa (95% CI)	Observed agreement (*n*/*n*, 95% CI)	PPV % (95% CI)	NPV % (95% CI)	Kappa (95% CI)	Observed agreement (*n*/*n*, 95% CI)	PPV % (95% CI)	NPV % (95% CI)	Kappa (95% CI)
Disease stage: stage I/II vs III/IV	0.91 (62/68, 0.82–0.96)	0.81 (0.62–0.94)	0.98 (0.87–1.00)	0.81 (0.67–0.95)	0.97 (66/68, 0.90–0.99)	0.92 (0.74–0.99)	1.00 (0.92–1.00)	0.94 (0.85–1.00)	0.97 (66/68, 0.90–0.99)	0.92 (0.74–0.99)	1.00 (0.92–1.00)	0.94 (0.85–1.00)
Disease stage: full stage (I/II/III/IV)	0.88 (60/68, 0.78–0.94)				0.96 (65/68, 0.88–0.98)				0.97 (66/68, 0.90–0.99)			
All sites combined	0.97 (854/884, 0.95–0.99)	0.98 (0.92–0.99)	0.98 (0.96–0.99)	0.92 (0.89–0.95)	0.99 (865/884, 0.97–0.99)	0.98 (0.96–0.99)	0.99 (0.96–1.00)	0.95 (0.93–0.98)	1.00 (873/884, 0.98–1.00)	0.99 (0.97–1.00)	1.00 (0.99–1.00)	0.97 (0.95–0.99)
All nodal sites combined	0.97 (653/680, 0.94–0.98)	0.99 (0.86–1.00)	0.98 (0.94–0.99)	0.91 (0.88–0.95)	0.98 (661/680, 0.96–0.99)	0.98 (0.96–0.99)	0.99 (0.94–1.00)	0.94 (0.91–0.97)	0.99 (669/680, 0.97–1.00)	0.99 (0.96–1.00)	1.00 (0.99–1.00)	0.97 (0.94–0.99)
All extra-nodal sites combined	0.99 (201/204, 0.96–1.00)	0.91 (0.71–0.98)	0.99 (0.96–1.00)	0.93 (0.84–1.00)	1.00 (204/204, NA)	1.00 (NA)	1.00 (NA)	1.00 (NA)	1.00 (204/204, NA)	1.00 (NA)	1.00 (NA)	1.00 (NA)
Nodal sites												
Cervical	0.97 (66/68, 0.90–0.99)	0.97 (0.89–1.00)	1.00 (0.40–1.00)	0.78 (0.50–1.00)	0.97 (66/68, 0.90–0.99)	0.97 (0.89–1.00)	1.00 (0.40–1.00)	0.78 (0.50–1.00)	0.99 (67/68, 0.92–1.00)	0.98 (0.91–1.00)	1.00 (0.48–1.00)	0.90 (0.71–1.00)
Axillary	0.96 (65/68, 0.88–0.98)	1.00 (0.82–1.00)	0.94 (0.83–0.99)	0.90 (0.78–1.00)	0.96 (65/68, 0.88–0.98)	1.00 (0.82–1.00)	0.94 (0.83–0.99)	0.90 (0.78–1.00)	0.97 (66/68, 0.90–0.99)	1.00 (0.83–1.00)	0.96 (0.86–1.00)	0.93 (0.84–1.00)
Infraclavicular	1.00 (68/68, 0.95–1.00)	1.00 (0.85–1.00)	1.00 (0.92–1.00)	1.00 (NA)	1.00 (68/68, 0.95–1.00)	1.00 (0.85–1.00)	1.00 (0.92–1.00)	1.00 (NA)	1.00 (68/68, 0.95–1.00)	1.00 (0.85–1.00)	1.00 (0.92–1.00)	1.00 (NA)
Mediastinal	0.99 (67/68, 0.92–1.00)	1.00 (0.94–1.00)	0.83 (0.36–1.00)	0.90 (0.71–1.00)	0.99 (67/68, 0.92–1.00)	1.00 (0.94–1.00)	0.83 (0.36–1.00)	0.90 (0.71–1.00)	1.00 (68/68, 0.95–1.00)	1.00 (0.94–1.00)	1.00 (0.48–1.00)	1.00 (NA)
Hilar	0.93 (63/68, 0.93–0.97)	0.96 (0.82–1.00)	0.88 (0.73–0.96)	0.82 (0.69–0.96)	0.97 (66/68, 0.90–0.99)	1.00 (0.88–1.00)	0.95 (0.82–0.99)	0.94 (0.86–1.00)	0.97 (66/68, 0.90–0.99)	1.00 (0.88–1.00)	0.95 (0.82–0.99)	0.94 (0.86–1.00)
Para-aortic	0.93 (63/68, 0.93–0.97)	0.94 (0.70–1.00)	0.92 (0.81–0.98)	0.81 (0.65–0.97)	0.96 (65/68, 0.88–0.98)	1.00 (0.79–1.00)	0.94 (0.84–0.99)	0.88 (0.76–1.00)	0.97 (66/68, 0.90–0.99)	1.00 (0.80–1.00)	0.96 (0.87–1.00)	0.92 (0.82–1.00)
Spleen	0.90 (61/68, 0.80–0.95)	0.83 (0.52–0.98)	0.91 (0.80–0.97)	0.68 (0.46–0.90)	0.90 (61/68, 0.80–0.95)	0.83 (0.52–0.98)	0.91 (0.80–0.97)	0.68 (0.46–0.90)	0.94 (64/68, 0.86–0.98)	0.87 (0.60–0.98)	0.96 (0.87–1.00)	0.83 (0.67–0.99)
Mesenteric	0.97 (66/68, 0.90–0.99)	1.00 (0.48–1.00)	0.97 (0.89–1.00)	0.82 (0.57–1.00)	1.00 (68/68, 0.95–1.00)	1.00 (0.59–1.00)	1.00 (0.94–1.00)	1.00 (NA)	1.00 (68/68, 0.95–1.00)	1.00 (0.59–1.00)	1.00 (0.94–1.00)	1.00 (NA)
Para-iliac	0.99 (67/68, 0.92–1.00)	1.00 (0.66–1.00)	0.98 (0.91–1.00)	0.94 (0.82–1.00)	0.99 (67/68, 0.92–1.00)	1.00 (0.66–1.00)	0.98 (0.91–1.00)	0.94 (0.82–1.00)	1.00 (68/68, 0.95–1.00)	1.00 (0.69–1.00)	1.00 (0.94–1.00)	1.00 (NA)
Inguinal femoral	1.00 (68/68, 0.95–1.00)	1.00 (0.16–1.00)	1.00 (0.95–1.00)	1.00 (NA)	1.00 (68/68, 0.95–1.00)	1.00 (0.16–1.00)	1.00 (0.95–1.00)	1.00 (NA)	1.00 (68/68, 0.95–1.00)	1.00 (0.16–1.00)	1.00 (0.95–1.00)	1.00 (NA)
Extra-nodal sites												
Abdominal	0.99 (67/68, 0.92–1.00)	1.00 (0.16–1.00)	1.00 (0.95–1.00)	NA	1.00 (68/68, 0.95–1.00)	1.00	NA	1.00 (NA)	1.00 (68/68, 0.95–1.00)	1.00 (0.16–1.00)	1.00 (0.95–1.00)	1.00 (NA)
Thoracic	1.00 (68/68, 0.95–1.00)	1.00 (0.74–1.00)	1.00 (0.94–1.00)	1.00 (NA)	1.00 (68/68, 0.95–1.00)	1.00 (0.74–1.00)	1.00 (0.94–1.00)	1.00 (NA)	1.00 (68/68, 0.95–1.00)	1.00 (0.74–1.00)	1.00 (0.94–1.00)	1.00 (NA)
Bone marrow	0.97 (66/68, 0.90–0.99)	0.90 (0.55–1.00)	0.98 (0.91–1.00)	0.88 (0.72–1.00)	1.00 (68/68, 0.95–1.00)	1.00 (0.69–1.00)	1.00 (0.94–1.00)	1.00 (NA)	1.00 (68/68, 0.95–1.00)	1.00 (0.69–1.00)	1.00 (0.94–1.00)	1.00 (NA)

*NA* not applicable

*Intrinsic MRI = consensus MRI DWI after removal of reader errors

**Table 5:** Discrepancies in stage between WB-MRI with and without DWI compared to the FDG-PET/CT-based reference standard

Patient	Stage			Discrepancy	Improved staging by addition of DWI
	Consensus MRI without DWI	Consensus MRI with DWI	FDG-PET/CT-based reference standard		
*1*	*3*	*4*	*4*	*Bone marrow disease was not seen on T1-weighted and T2-weighted images and was detected by the addition of DWI*	*Yes*
2	4	4	4	Hilar was scored negative without DWI due to size criteria and positive with DWI. The reference standard scored positive as well	Discrepancy did not influence disease stage
3	2	2	2	Hilar was scored negative without DWI due to size criteria and positive with DWI. The reference standard scored positive as well	Discrepancy did not influence disease stage
4	2	2	2	Hilar was scored negative without DWI due to size criteria and positive with DWI. The reference standard scored positive as well	Discrepancy did not influence disease stage
*5*	*2*	*3*	*3*	*The stations para-aortic and mesenteric were scored false negative at consensus MRI without DWI due to size criteria. Both stations were scored positive with the addition of DWI. The reference standard scored both stations positive as well*	*Yes*
*6*	*3*	*2*	*2*	*Para-aortic was scored false positive at consensus MRI without DWI. At consensus MRI with DWI no para-aortic disease was detected, similar to the reference standard*	*Yes*
*7*	*4*	*2*	*2*	*Bone marrow was scored false positive at consensus MRI without DWI. At consensus MRI with DWI, no bone marrow disease was detected, similar to the reference standard*	*Yes*
*8*	*4*	*2*	*2*	*Liver was scored false positive at consensus MRI without DWI. At consensus MRI with DWI, no liver disease was detected, similar to the reference standard*	*Yes*
9	4	4	4	Mesenteric was scored negative without DWI due to size criteria and positive with DWI. The reference standard scored positive as well.	Discrepancy did not influence disease stage

*Italic:* patients in whom staging improved with the addition of DWI to the MRI sequences

Sensitivity and specificity for staging paediatric HL using consensus WB-MRI without DWI were 96% (95% CI 0.78–1.00) and 89% (95% CI 0.76–0.96) respectively, whereas for WB-MRI including DWI, the sensitivity and specificity increased to 100% (95% CI 0.85–1.00) and 96% (95% CI 0.85–0.99) respectively.

### Intrinsic WB-MRI versus FDG-PET/CT-based reference standard

**Fig. 4:**
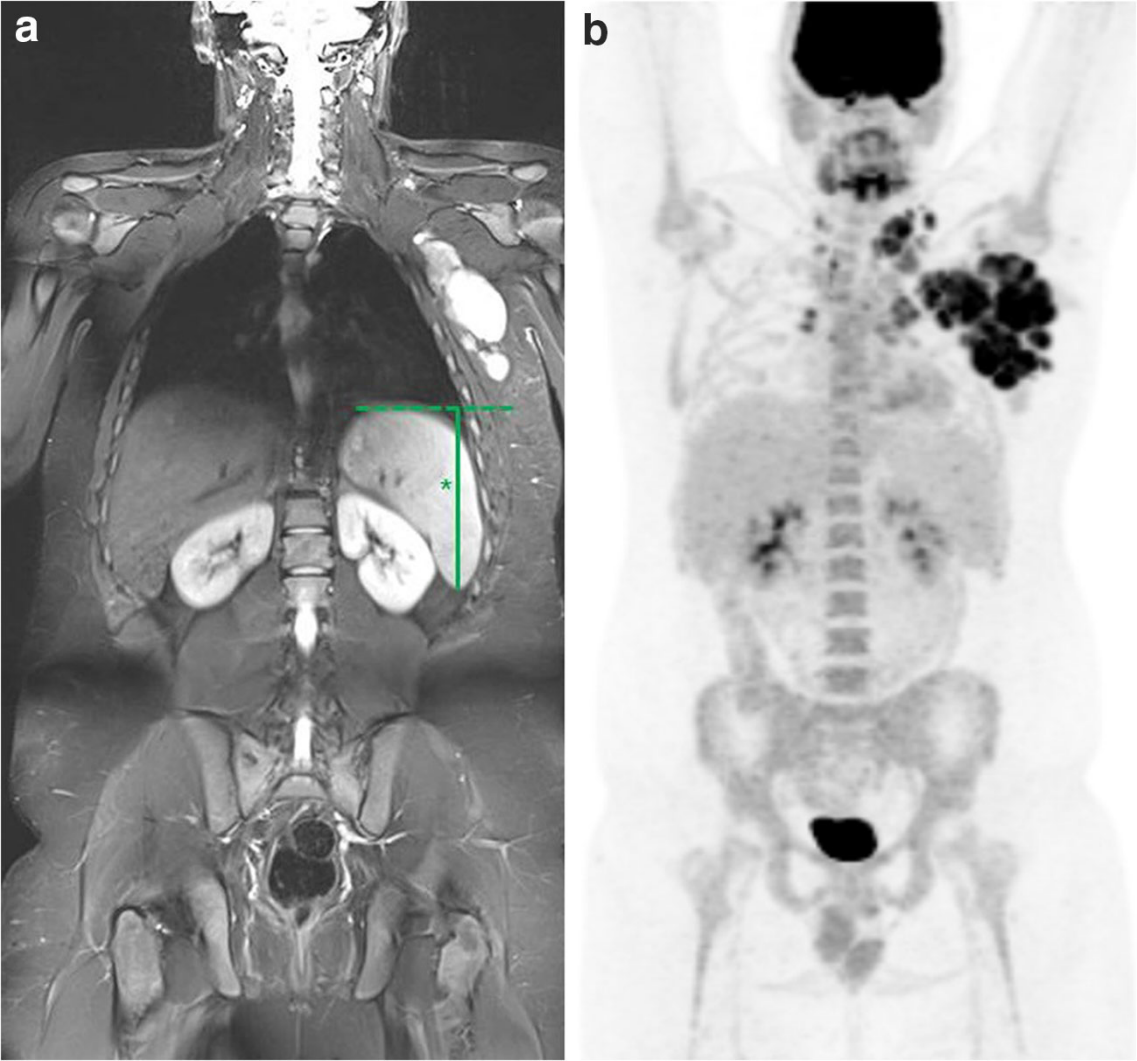
A 17-year-old boy with stage II Hodgkin lymphoma. Example of upstaging by WB-MRI due to enlargement of the spleen without focal lesions. Coronal T2-weighted WB-MRI (**a**) shows enlargement of the spleen without focal lesions (*size = 14.1 cm). Coronal maximum intensity projection (MIP) of the FDG-PET (**b**) demonstrates no involvement of the spleen. The involved lymph node stations are cervical, axillary, infraclavicular, mediastinal and hilar (**b**)

For intrinsic WB-MRI (consensus WB-MRI including DWI without reader errors), concordance in disease stage was reached in 66/68 patients (97.1%). Two patients were over-staged (stage 3 versus stage 2) due to enlargement of the spleen without focal lesions and without disease presence at FDG-PET/CT (Fig. 4). Kappa agreement was excellent (*κ* 0.94, 95% CI 0.85–1.00). Sensitivity and specificity of the intrinsic MRI for staging were 100% (95% CI 0.85–1.00) and 96% (95% CI 0.85–0.99).

Table 4 shows the observed agreement rate, PPV, NPV and Cohen’s kappa values for all assessed disease sites. When comparing the intrinsic WB-MRI with the reference standard, observed agreement, PPV, NPV and kappa agreements were 99%, 0.99, 1.00 and 0.97, respectively, for nodal disease. Agreement was 100% for extra-nodal disease. There were 11 discrepant disease sites. WB-MRI was false positive in 3 nodal sites (cervical (1) and spleen (2)) and false negative in 8 nodal sites (axillary (2), hilar (2), para-aortic (2) and spleen (2)) compared to the reference standard.

## Discussion

This prospective, multicentre, international study in 68 children with newly diagnosed Hodgkin lymphoma compared the concordance of WB-MRI with and without DWI to an FDG-PET/CT-based reference standard for the initial staging of paediatric HL.

Results show a good inter-observer agreement between the WB-MRI readers for both nodal and extra-nodal staging. Previous studies found comparable agreements [16, 17]. The lymph node stations with the highest amount of discrepancies between WB-MRI DWI readers were infraclavicular, hilar and mesenteric. This was mostly due to labelling errors. Mesenteric lymph nodes were scored as para-aortic; infraclavicular lymph nodes were sometimes mistaken for cervical lymph nodes, and hilar lymph nodes were marked as mediastinal or vice versa. In most cases, these labelling errors did not affect the conclusions on determining disease stage. Although motion artefacts were present in part of the WB-MRI scans (mainly cardiac or respiratory motion artefacts), these artefacts did not cause (labelling) errors.

For disease stage, the agreement between the reference standard and the intrinsic WB-MRI was 97.1%. This sounds promising, but for the two discrepant cases, this would have had relevant implications for treatment planning in clinical practice. According to WB-MRI, both patients would have been staged stage 3, implicating advanced disease and thus a more intensive treatment scheme, whereas the FDG-PET/CT-based reference standard staged both patients stage 2 which is considered limited disease with, also due to the absence of B-symptoms in both patients, a less intensive treatment regime. In both cases, the discrepancy was caused by an enlargement of the spleen without being FDG-PET/CT positive. The size criterion for WB-MRI thus caused an inaccuracy for detecting disease presence in the spleen. In clinical practice, most patients receive an ultrasound examination at first presentation as well. This provides extra information regarding splenic involvement which was not considered for this study. With the addition of DWI to the WB-MRI reading, the agreement on disease stage improved for five patients, as is shown in Table 5. This difference in agreement on disease stage was statistically significant (*p* = 0.036).

The concordance between intrinsic WB-MRI DWI and the FDG-PET/CT-based reference standard was 100% for extra-nodal disease. For nodal disease, the concordance was 99%. The agreements found in this study resemble those found in recent literature, as Latifoltojar et al recently reported 99% concordance for nodal disease and > 99% for extra-nodal disease for their WB-MRI reading after removal of perceptual errors [17]. The 100% overall agreement in determining extra-nodal disease that was found for both WB-MRI DWI and intrinsic WB-MRI DWI implies that no lung lesions have been missed by WB-MRI DWI. Detection of lung lesion is the most important reason to also perform a separate CT examination nowadays, which causes extra exposure to ionizing radiation.

When assessing the separate lymph node stations, the agreement between intrinsic WB-MRI and the reference standard was good to excellent for all stations for both WB-MRI with and without DWI. With the addition of DWI, the agreement with the reference standard remained the same or improved for all stations. Main improvements were seen for the hilar (*κ* 0.88 to *κ* 0.94), para-aortic (*κ* 0.81 to *κ* 0.88), mesenteric (*κ* 0.82 to *κ* 1.00) and bone marrow (*κ* 0.88 to *κ* 1.00) stations. Therefore, in line with our previous results, DWI was mainly of additional value in the abdominal lymph node stations [28].

There are a few limitations of this study that need to be addressed. An unblinded expert panel created the FDG-PET/CT-based reference standard. This was performed in a consensus reading and with the availability of all collected data. Due to the lack of a true gold standard, this was the best available option to form a reference standard. This method for creating a reference standard when no true gold standard is available has been used by others as well [16, 17, 29]. Since the expert panel used all available data, the WB-MRI data and reference standard were not completely independent of each other. However, this method does resemble clinical practice in which final decisions are made in consensus. For this study, the differences between WB-MRI and the reference standard might be underestimated due to this design.

Furthermore, all reader errors from the WB-MRI DWI reading were removed to create the intrinsic WB-MRI dataset. Although this probably provides the best available WB-MRI results, reader errors are of course part of daily clinical practice as well and the intrinsic WB-MRI is thus likely be an overestimation of reality. However, it can also be argued that the intrinsic MRI does resemble clinical practice in the best way possible since in clinical practice all patients are discussed in multidisciplinary meetings where not only imaging results, but clinical, histological and laboratory findings are considered as well. The intrinsic MRI showed only small increases in agreements when compared to WB-MRI including DWI. Therefore, the overestimation of the intrinsic WB-MRI accuracy seems to be limited.

In contrary to the WB-MRI reading, the FDG-PET/CT reading was performed by only one experienced reader as a result of which no inter-observer agreement could be determined. This limitation was overcome by the expert panel (including a nuclear medicine physician), who created the FDG-PET/CT-based reference standard by assessing all available information.

Finally, the focus of this study was the initial staging of paediatric HL. During the course of diagnosis, treatment and follow-up, children with HL are exposed to multiple imaging examinations. As a consequence, the amount of administered ionizing radiation can accumulate to significant levels, especially when considering that contrast-enhanced CT (CE-CT) is still widely used for imaging paediatric HL. The performance of WB-MRI in other phases of the disease process, such as response evaluation and restaging, might differ from the performance for staging. Although response assessment has recently been addressed in the literature by a few studies, the use of WB-MRI for both response evaluation and restaging does need further investigation [17, 30].

To conclude, inter-observer agreement of WB-MRI DWI was good for both nodal and extra-nodal staging and for determining disease stage. The addition of DWI to the WB-MRI protocol in staging of paediatric HL improved staging agreement with the FDG-PET/CT-based reference standard. Concordance between intrinsic WB-MRI DWI and the FDG-PET/CT-based reference standard was excellent, but did not reach 100%, due to discrepancies in staging of splenic involvement.

## Electronic supplementary material

ESM 1 (DOCX 23.1 kb)

## Abbreviations

CE-CT: Contrast-enhanced CT
DWI: Diffusion-weighted imaging
FDG-PET/CT: 18F-Fluorodeoxyglucose positron emission tomography/computed tomography
HL: Hodgkin lymphoma
IQR: Interquartile range
mSV: Millisievert
NPV: Negative predictive value
PPV: Positive predictive value
SD: Standard deviation
WB-MRI: Whole-body magnetic resonance imaging
