# Supplementary material for: Whole-body MRI versus an FDG-PET/CT-based reference standard for staging of paediatric Hodgkin lymphoma: a prospective multicentre study
Source: Eur Radiol. 2020 Sep 3;31(3):1494–504. doi: 10.1007/s00330-020-07182-0 (PMC7880958; doi:10.1007/s00330-020-07182-0)
Supplement: Supplementary file 1 — (DOCX 23.1 kb) [file 330_2020_7182_MOESM1_ESM.docx]

**Supplementary material**

WB-MRI protocol

Whole-body MRI was performed using a 1.5-T system (Philips Healthcare or Siemens or GE Medical Systems. Choice of appropriate coils for signal reception depended on the available MR system and hardware in each participating centre.

In this protocol, whole-body MR image acquisition was defined as image acquisition from the top of the head to the upper thigh, which is comparable to the scan range commonly applied in PET/CT imaging. The actual scan range varied slightly according to applied MR hardware and according to height of the patient, but minimally included the upper cervical region to the inguinal/femoral lymph node regions.

First, coronal whole-body T1-weighted (T1W) and short inversion time inversion recovery (STIR) images were acquired, using the built-in body coil or the whole-body surface coil design for signal reception. Images were acquired under free breathing, except for the stations covering the chest and abdomen, which were acquired using breath holding (T1W) or respiratory triggering (STIR). Second, axial diffusion-weighted images of the head/neck, chest, abdomen, and pelvis were acquired under free breathing.

Data reconstruction was performed on an MRI image analysis workstation (e.g. View Forum, Philips Medical Systems or Leonardo, Siemens or Advantage Windows Workstation, GE Medical Systems). Seamless coronal whole-body T1W and STIR images were created by merging separately acquired stations using software implemented in the standard operating console. Axial diffusion-weighted images were first coronally reformatted with a slice thickness/gap of both 3.5/0mm and 7/0mm, and then merged to create seamless coronal whole-body diffusion-weighted images. In addition, seamless 3D maximum intensity projection (MIP) reconstructions were created (36 slices x 5 degrees angle in axial plane, rotating around cranio-caudal axis). Whole-body diffusion-weighted images were displayed using greyscale inversion.

The total duration of the examination was about 50-60 minutes, including patient preparation time.

FDG-PET/CT protocol

FDG-PET/CT (Biograph 16 PET-CT or Biograph 40 Truepoint PET-CT, Siemens Healthcare; Gemini TOF PET- CT or Allegro, Philips Healthcare; spatial resolution around 5 mm at full width at half maximum) was performed after at least six hours of fasting. Blood glucose levels were measured and had to be below 11 mmol/L for the procedure to continue. 2 MBq/kg body weight of FDG was administered in every patient. Image acquisition took place 60 minutes after FDG administration.

Low-dose CT scanning was performed with the following settings: 120 kV, 26-30 mAs, 0.8-s tube rotation time, pitch of 1.2, and 1.5-mm slice width (reconstructed to contiguous 5-mm axial slices to match the section thickness of the PET images). After the low-dose CT, PET scanning was performed from mid-thighs to the base of the skull in five- six bed positions, with three minutes per bed position. Low-dose CT was used for attenuation correction of PET data.

PET images were reconstructed using an ordered-subsets expectation maximization algorithm for 14 subsets and four iterations. The image reconstruction matrix was 128 × 128.

The duration of the examination was approximately 90 minutes including the 60 minute waiting time between admission of FDG and the scan.

Supplementary table 1. WB-MRI scan parameters

|  | 1.5 T | | |
| --- | --- | --- | --- |
|  | Pulse sequence | | |
|  | T1W TSE | T2W STIR | DWIBS STIR |
| Orientation | Coronal | Coronal | Axial/coronal |
| Repetition time (ms) | 518-637 | 2826-6070 | 3200-9754 |
| Echo time (ms) | 7-27 | 44-81 | 61-80 |
| Inversion time (ms) | - | 165 | 180 |
| Slice thickness/ slice gap (mm) | 6/1 | 6/1 | 6/0 |
| Number of slices per station | 30 | 30 | 60 |
| Field of view (mm2) | 530 x 265 | 530 x 265 | 450 x 360 |
| Acquisition matrix | 384 x 256 | 336 x 267 | 128 x 81 |
| B-values (s/mm2) | - | - | 0, 100, 800 |
| Number of signals averaged | 1 | 1-2 | 3-5 |
| Respiratory motion compensation | Free breathing, except for thorax and abdomen (breath holding) | Free breathing, except for thorax and abdomen (respiratory triggering) | Free breathing |

Supplemental table 1: WB-MRI detailed imaging parameters. Parameters vary depending on the system used by the participating centres (Philips, Siemens or GE). *DWIBS, diffusion weighted imaging with background body signal suppression; STIR, short inversion time inversion recovery; T, Tesla; T1W, T1-weighted; T2W, T2-weighted; TSE, turbo spin echo.*
